# Automated Detection of Symptomatic Autonomic Dysreflexia Through Multimodal Sensing

**DOI:** 10.1109/JTEHM.2019.2955947

**Published:** 2020-01-20

**Authors:** Shruthi Suresh, Bradley S. Duerstock

**Affiliations:** 1 Weldon School of Biomedical EngineeringPurdue University 311308 West LafayetteIN47907USA; 2 School of Industrial EngineeringPurdue University 311308 West LafayetteIN47907USA

**Keywords:** Machine learning, physiological telemonitoring, spinal cord injuries, support vector machines, wearable computer

## Abstract

Objective: Autonomic Dysreflexia (AD) is a potentially life-threatening syndrome which occurs in individuals with higher level spinal cord injuries (SCI). AD is caused by triggers which can lead to rapid escalation of pathophysiological responses and if the trigger is not removed, AD can be fatal. There is currently no objective, non-invasive and accurate monitoring system available to automatically detect the onset of AD symptoms in real time in a non-clinical setting. Technology or Method: We developed a user-independent method of symptomatic AD detection in real time with a wearable physiological telemetry system (PTS) and a machine learning model using data from eleven participants with SCI. Results: The PTS could detect onset of AD symptoms with an average accuracy of 94.10% and a false negative rate of 4.89%. Conclusions: The PTS can detect the onset of the symptoms AD with high sensitivity and specificity to assist people with SCIs in preventing the occurrence of AD. It would enable persons with high level SCIs to be more independent and pursue vocational activities while granting continuous medical oversight. Clinical Impact: The PTS could serve as a supplementary tool to current solutions to detect the onset of AD and prepare individuals who are newly injured to be better prepared for AD episodes. Moreover, it could be translated into a system to encourage individuals to practice better healthcare management to prevent future occurrences.

## Graphical abstract

In this paper we present a highly accurate and sensitive, user-independent Physiological Telemetry System which can detect the onset of autonomic dysreflexia symptoms.

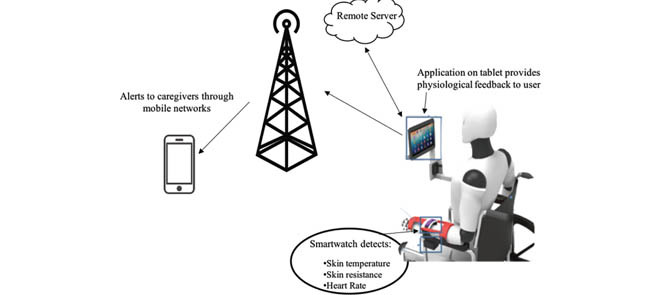

## I. Introduction

Autonomic dysreflexia (AD) is a potentially life threating syndrome which occurs in roughly 70% of all persons with a spinal cord injury (SCI) above the T6 level [1]. AD is caused by hyperactivity of the sympathetic nervous system initiated by noxious or innocent stimuli below the level of injury. These include urinary tract infections, impacted bowels, wounds, or sexual activity [2], [3]. Notable pathophysiological responses when AD occurs are paroxysmal hypertension of at least 20 mm Hg [4]–[6], the onset of sweating above the level of injury, bradycardia and/or tachycardia, and changes in skin temperature [7], [8]. AD causes mild to debilitating symptoms including headaches, acute anxiety, cold and clammy skin, shivering, blurred vision, and facial flushing. If the trigger is not removed, AD can escalate rapidly leading to systolic hypertension of over 200 mmHg, seizures, hemorrhagic strokes or even death [9], [10].

However, being familiar with the symptoms and the triggers of AD remains the prescribed approach by therapists and physicians [11] to preventing the onset or escalation of AD symptoms [12]. Outside of clinical settings, AD is not routinely monitored by healthcare professionals due to the lack of a reliable, wearable monitoring device. Although blood pressure measurements are used to diagnose AD clinically, this method is inadequate for continuous monitoring for the presence of AD. Therefore, it is imperative to utilize other sympathetic sensors to detect the onset of AD in a community setting.

A wearable physiological telemetry system (PTS) was developed to collect multimodal sympathetic physiological data and automatically detect the onset of AD symptoms by training a machine learning model using data from tetraplegics [13], [14]. The PTS uses sensors for galvanic skin response (GSR), heart rate, and skin temperature on a wrist-worn smartwatch connected to a tablet to positively confirm the presence of AD symptoms with participants with SCI who have learned their unique symptoms of AD over the course of years.

Multiple Support Vector Machine (SVM) models were trained using the collected data of human participants with chronic SCI for AD detection. An optimal AD detection model was determined based on performance metrics such as accuracy and sensitivity. We validated the system in real-world community settings through its performance in detecting the onset of AD symptoms in naïve participants with tetraplegia with 94% accuracy.

The paper is structured as follows. Section II presents the current work in detection of AD. Section III explains in detail of the development of the PTS. Section IV presents an evaluation of the developed PTS with results of the evaluation shown in Section V. Section VI and VII present the discussions and conclusions.

## II. Related Work

Currently, the management and prevention of AD fundamentally begins with behavioral changes. Acute episodic AD can be managed by changing posture, loosening or removing tight clothing, shoes or compression stockings [15]. However, newly injured individuals often have to experience AD symptoms several times before familiarizing themselves with the triggers and the corresponding symptoms. This repeated onset of AD without sufficient knowledge of proper management techniques could lead to severe health consequences [2]. The prevalence of AD often increases according to the level and severity of the SCI, predominantly high-level tetraplegics with complete injuries [16], [17]. While AD has been reported to occur between 5 to 40 times per day, the frequency and severity of AD are dependent on the individual [18]. These factors make it difficult to predict the onset of symptoms of AD except after years of recognizing one’s own AD symptoms.

AD most often presents between two to six months’ post injury [5], [7]. However, almost 90% of all newly injured SCI patients are discharged to their homes under the care of family members and home healthcare providers after receiving only approximately 3 weeks of in-hospital rehabilitation [19]. Thus, some individuals may not experience AD until they are discharged from the hospitals, leaving them to learn to recognize their symptoms outside of clinical settings. In addition, only 41% of persons with chronic SCIs and their family had heard of AD, while 22% of individuals with SCI reported symptoms consistent with unrecognized AD [20]. This lack of familiarity makes AD a dangerous medical issue for newly injured individuals.

Within clinical settings, onset of AD is manually identified through ambulatory blood pressure monitoring (ABPM) systems which can regularly measure systolic and diastolic blood pressure [9], [21]. ABPM systems have been used to develop software to perform automated detection and evaluation of cardiovascular autonomic dysfunction after SCI [5] in clinical settings. They have also been used to detect the changes in BP due to AD in urodynamic settings [22]. However, continuously monitoring blood pressure (BP) using the ABPM is not practical for long-term use in a community setting for daily use because it restricts individuals’ activities and can be affected by strong movements, such as wheeling or transferring [23], [24]. Other ANS responses are also well known to reliably change due to AD, in particular GSR to detect pathological sweating above the level of injury and irregular changes in heart rate [14], [25], [26].

There is currently no system which can detect the onset of AD symptoms in real-time in a non-clinical, community setting. Through this paper, we studied the ability of the PTS to be used in a community setting to evaluate its ability to detect the onset of symptomatic presentation of AD in real-time.

## III. Development of the Physiological Telemetry System

**FIGURE 1.**
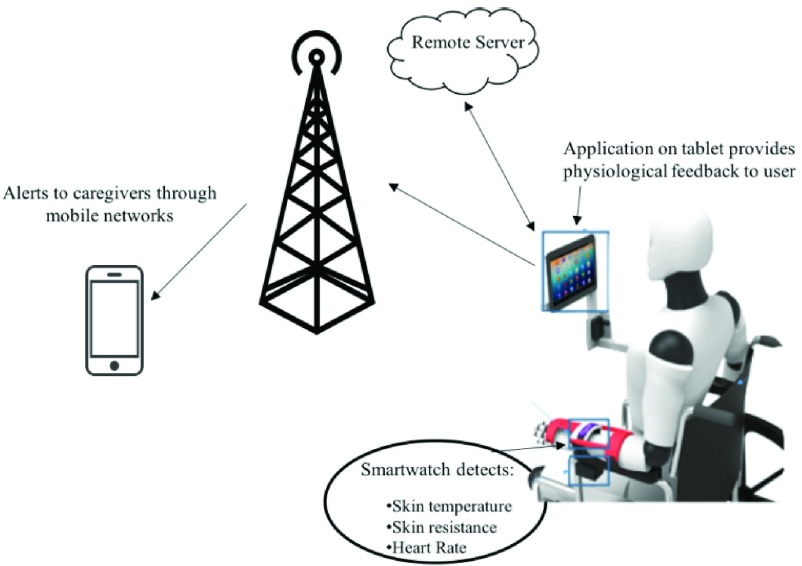
Overview of the physiological telemetry system used to collect data and predict onset of AD symptoms.

**FIGURE 2.**
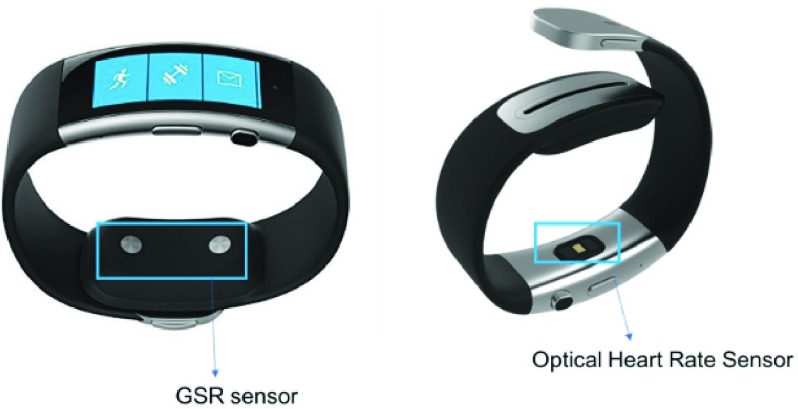
The Microsoft band and its sensors for GSR, heart rate, and skin temperature.

The Physiological Telemetry System (PTS) was previously developed to collect the physiological data and detect the onset of AD (Fig. 1) [14], [27]. The PTS is comprised of non-invasive, wearable sensors for skin temperature, GSR and heart rate, whose data are wirelessly streamed to a mobile application over the internet. These sensors are available in the Microsoft® Band (MS Band) (Fig. 2), a wrist-worn smartwatch. It was chosen for its wearability, ability to develop custom applications and controlled sampling rate [28]. A mobile application was developed and deployed on an Android® tablet via Bluetooth, to receive data from the watch and alert the users when AD is detected.

### A. Study Participants

Patients with cervical and thoracic level injuries were recruited from the outpatient program in the Rehabilitation Hospital of Indiana, Indianapolis, Indiana, United States. The inclusion criteria for the study were (i)18–70 years of age (ii) a SCI above the sixth thoracic segment (T6), (iii) injured for at least 3 years prior to participation, (iv) familiar with their symptoms of AD and management strategies prior to enrollment in study and (v) sufficient motor ability in upper limbs to manipulate a smartphone or tablet.

Participants were excluded if they experienced any health problems unrelated to SCI, such as chronic heart conditions, diabetes, unstable psychiatric condition, or any cognitive dysfunction.

Seven participants with cervical and upper thoracic injuries were screened and recruited (Table 1) for development of the system. The mean age of the participants was 38.8 ± 8.1, with 6 male and 1 female participants which is similar to epidemiology of SCI. All subjects had a cervical SCI. 4 participants had complete SCIs resulting in tetraplegia and 3 had incomplete injuries. Prior to their participation in the study, the subjects had been injured for 19.0 ± 7.8 years. All study protocols were approved by the Institutional Review Board of Purdue University. Prior to the study, informed consent was obtained from all the participants.

**TABLE 1:** Characteristics of Study Participants

Participant ID	Age	Gender	Level of Injury	Completeness (C/I)	Years since injury
Participant 1	45	M	C4/C5	C	26
Participant 2	26	M	C5/C6	C	4
Participant 3	28	M	C5/C6	I	12
Participant 4	48	M	C6/C7	C	25
Participant 5	42	M	C6/C7	C	19
Participant 6	45	M	C6/C7	I	27
Participant 7	38	F	C6/C7	I	20

### B. Developing the Machine Learning Model

#### 1) Data Collection

**FIGURE 3.**
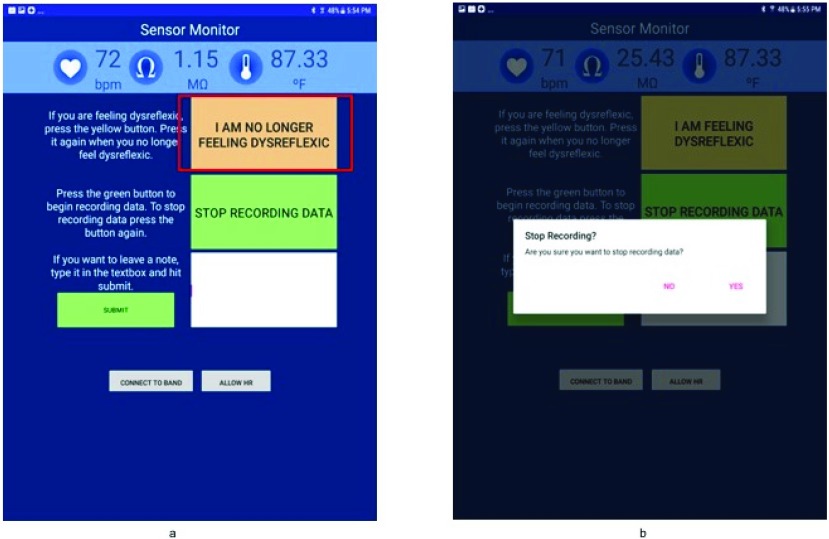
The Android sensor recorder application with features for the user to; (a) report the onset of AD, (b) stop recording data.

The seven participants (Participants 1–7) were asked to use the PTS every day for a duration of one week for at least 8 hours each day (10AM-6PM) while performing their typical activities of daily life. During the study, participants were instructed to use an application developed for the PTS running on a mobile tablet to report any onset of AD symptoms they experienced. Participants used the interface shown in Fig. 3 to report the onset of AD symptoms, the severity of the AD and also stop recording data. For example, the participant presses the button “I am Feeling Dysreflexic” when they start to feel symptomatic of AD. The text on the button changes to “I am no longer feeling dysreflexic”. The data collected during this period of time is labeled as “onset of AD”.

In this study, we rely on the individuals’ ability to self-report symptoms of AD. This is supported by studies in which participants were asked to self-report AD symptoms which showed high correlation between self-reported frequency and the objectively assessed number of AD events [10].

All the collected data were transmitted wirelessly to a cloud-based server for the training of a machine learning model using SVM.

#### 2) Data Normalization

Due to differences in the magnitudes of the various physiological parameters in different participants, the data collected was standardized through a min-max method, which scales data in a range of 0 to 1. Normalization of input data eliminated the differences introduced by variance between users and made the machine learning model generalizable to the population. Each physiological parameter was normalized through this method. For example, as shown in (1), the skin resistance data for each participant SR_sub_ was normalized to SR_norm_ by subtracting the minimum SR_min_ and scaling it against the difference of SR_max_ and SR_min_.

\begin{equation*} {SR}_{norm}= \frac {SR_{sub}- {SR}_{min}}{SR_{max}- {SR}_{min}}\tag{1}\end{equation*}

#### 3) Training the SVM Model

In order to classify the physiological data, we used an SVM with a radial basis function (RBF) kernel. The SVM was chosen due to its ability to segregate interspersed data as well as ease of implementation. In prior work, we have established a higher performance of an SVM over other linear classifiers [13].

The normalized physiological data from all the subjects was randomized before being used to train the SVM. Each sample $x_{i}$, $i =1, \ldots, n$ consisted an N by three feature vector, where N is the length of the data in terms of time, and the time-series data from the three sensors of the MS Band serve as features. The SVM separates the data into two class labels, $y_{i} \in $ {+1, 0} wherein +1 represents the onset of AD symptoms and 0 represents a lack of AD symptoms.

**FIGURE 4.**
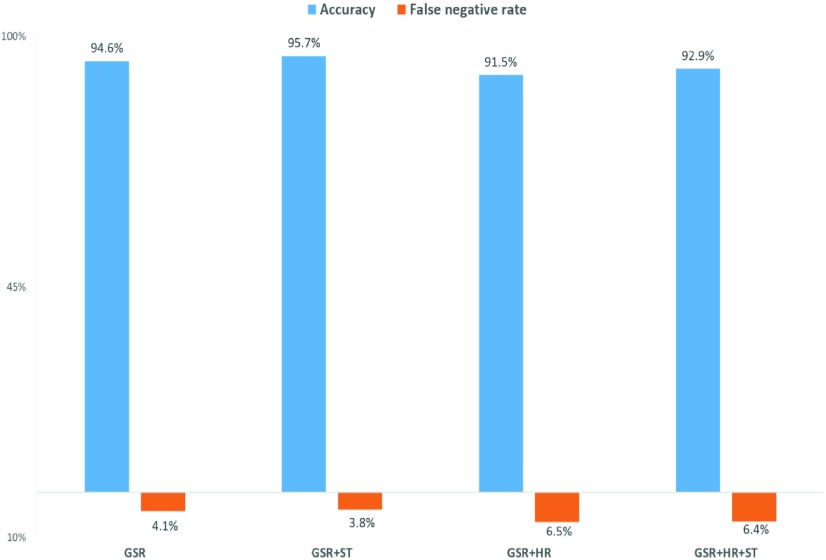
Percentage accuracy and false negative rate of the model using combinations of physiological parameters- GSR, heart rate (HR) and skin temperature (ST). The combination of GSR and ST was chosen as the optimal test model (N=7).

Four different models were trained using various combinations of physiological sensor data (GSR, skin temperature and heart rate) as features. A 5-fold cross validation was used on all the feature vectors to determine its accuracy, sensitivity and specificity of each model (Fig. 4). This ensures the development of a model which is less biased. The sensitivity of the machine learning model is its ability to correctly classify an AD event when it was self-reported by the individual while the specificity is the ability of the model to correctly classify a normal event as a non-AD event. The false negative rate (FN rate = 1-*sensitivity*) was the model’s incorrect classification of a self-reported AD event as normal, and the false positive rate (FP rate = 1-*specificity*) was when a normal event was misclassified as an AD event (Table 2).

**TABLE 2:** Representation of the Confusion Matrix for AD Detection

	Predicted AD	Predicted Non-AD
Actual AD	True Positive (TP)	False Negative (FN)
Actual Non-AD	False Positive (FP)	True Negative (TN)

Sensitivity: True positive rate; Specificity: True Negative rate

**TABLE 3:** Characteristics of Study Participants for Evaluation of the PTS

Participant ID	Age	Gender	Level of Injury	Completeness (C/I)	Years since injury
Participant 8	18	M	C5/C6	I	3
Participant 9	37	M	T1	I	15
Participant 10	29	M	C5	C	3
Participant 11	40	F	C6	C	6

#### 4) The Optimal SVM Model

**FIGURE 5.**
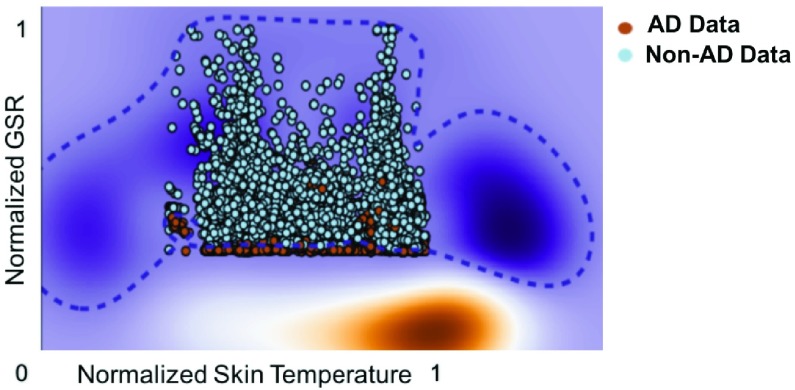
The “test” model developed with an RBF kernel distinguishes AD and non-AD data.

The model with the highest accuracy and lowest false negative rate was chosen as the optimal model for the detection of AD in real time. The results for the four models are shown in Fig. 4. When GSR and skin temperature were used as the primary parameters, the machine learning model developed was most accurate (95.7%). Therefore, this optimal model was chosen as the “test model” (Fig. 5) for real-time evaluation of the PTS’ ability to detect the onset of AD symptoms.

## IV. Evaluation of the PTS

In order to evaluate the ability of the optimal test model to detect the onset of AD symptoms without being prompted, naïve participants were recruited. Their data was not used in the training of the test model. The detection accuracy, sensitivity, specificity are used for the evaluation in addition to questionnaires about usability and comfort of the overall system.

### A. Study Participants

Four participants (Participants 8–11) were recruited for the evaluation of the PTS’ ability to identify and alert the users of onset of AD symptoms. The mean age of these participants was 31 ± 8.5, with 3 male and 1 female participant. They had been injured for 6.7 ± 4.9 years. All participants met the inclusion criteria and informed consent was obtained from all the participants.

### B. Experimental Procedure

**FIGURE 6.**
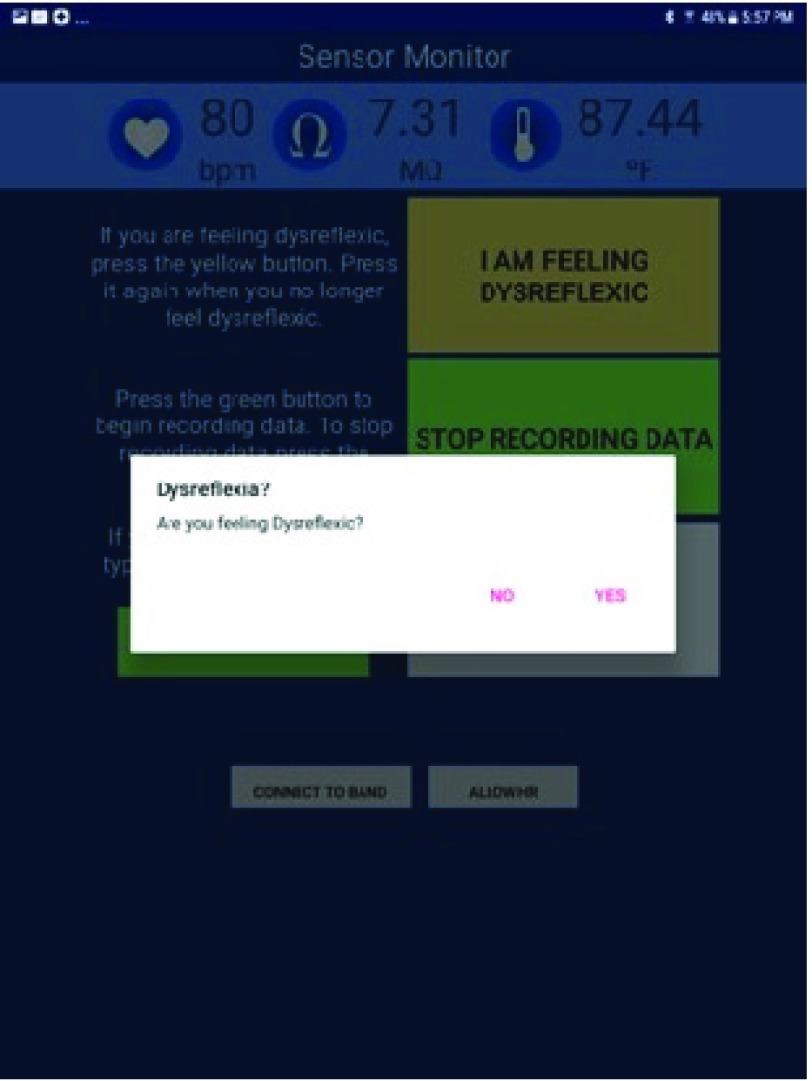
The Android sensor recorder application to verify the detection of AD symptoms by the PTS machine learning model.

The participants were instructed to use the PTS for 8 hours a day (10AM-6PM) daily for a week. In addition to self-reporting onset of AD symptoms, the participants were instructed to respond to prompts on the tablet when the machine learning model detected the onset of AD symptoms. Data from the MS Band was sent to a remote server running the machine learning model which determined if the incoming data represented a symptomatic AD event. If AD was detected, the mobile application alerted the user through a visual notification and sound (Fig. 6). The participants confirmed the presence of AD by clicking “YES” or “NO” to identify true or false positives. There is also a button for participants to self-report the onset of AD if the trained model did not detect the symptoms (false negative). The participants were also asked to complete a usability survey after their experiment over the week.

### C. Evaluating the Test Model

A confusion matrix was calculated to determine the overall accuracy, sensitivity and specificity of the machine learning algorithm with positive (AD detection) and negative (non-AD detection) class values [29]–[32] (Table 2). The model’s receiver operating characteristic (ROC) curve was calculated to visualize the performance of binary classifier and determine the Area Under the ROC curve (AUC-ROC) [32]. A high AUC-ROC shows a better performance of the test in detecting AD and a higher priority of false negatives. The Area Under the ROC (AUC-ROC) curve summarizes the performance in a single number. In a perfectly discriminatory test, the AUC-ROC is 1.0

### D. Assessing Usability of the System

At the end of the experiment, the participants were requested to assess the PTS using a System Usability Scale (SUS) for long-term AD monitoring [33]. The SUS is a 10-item questionnaire about the usability of the system and levels of agreement with ten statements are scored using a five-point Likert scale anchored with ‘strongly disagree’ (1) and ‘strongly agree’ (5). It measures aspects of usability such as perceived complexity, consistency and integration of the system. Scoring of the SUS allows a conversion of the evaluated scores to a range of 0 to 100. Outliers were removed through the interquartile method [34]. The validity, reliability and sensitivity of the SUS have been extensively evaluated and it has been found to be a reliable measure of usability [35], [36].

## V. Results of Evaluation

### A. Evaluating the Optimal Machine Learning Model

The performance of the most accurate “test model” with GSR and ST as primary physiological parameters was shown in Fig. 5. The average detection accuracy was 94.10% with a false positive rate of 4.89%.

When evaluated on the naïve participants whose data was excluded from the training of the model, the test model correctly identified AD (sensitivity) 95.11% of the time and correctly identified a non-dysreflexic state of being an AD episode (specificity) 93.81% of the time (Table 4).

**TABLE 4:** Evaluation Parameters Using the Test Model

Participant ID	Accuracy	Sensitivity	Specificity	False Positive Rate
Participant 8	87.17%	86.84%	98.80%	1.20%
Participant 9	99.49%	99.51%	96.90%	3.10%
Participant 10	99.70%	99.81%	87.10%	12.90%
Participant 11	90.03%	89.10%	97.62%	2.38%
Average	94.10%	93.81%	95.11%	4.89%

**FIGURE 7.**
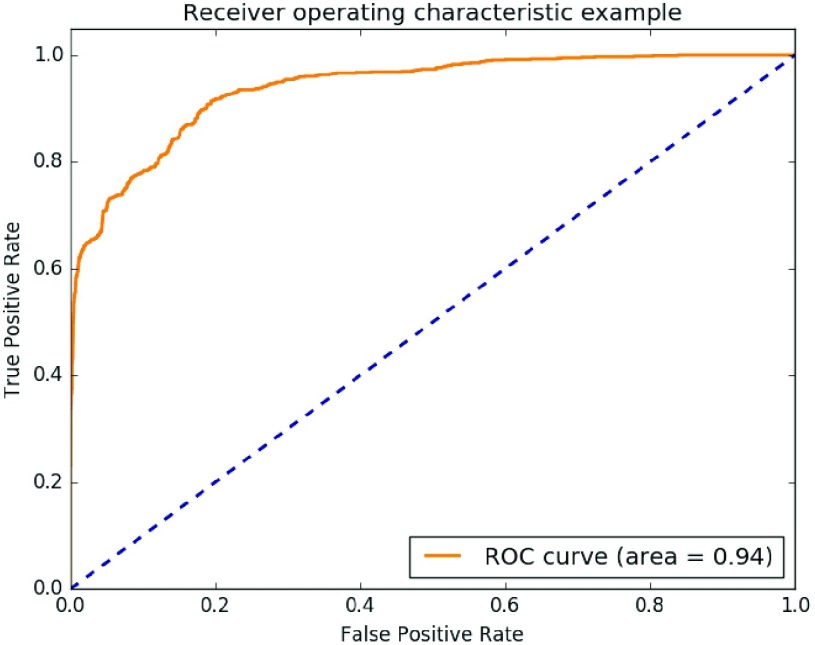
ROC Curve for the machine learning model for true and false positives.

A Receiver Operating Curve (ROC) curve was used to visualize the performance of the SVM model to truly identify AD (true positive) and mislabel normal events as AD (false positive) (Fig. 7). For the test model developed from a combination of GSR and skin temperature as primary parameters, this area was determined to be 0.94.

### B. Usability of the PTS

Based on SUS results from participants who received prompts for AD detection during the evaluation of the PTS, scored its usability at 86.2±9.7. Most of the participants stated that they would use the system frequently and would be able to use the system independently without much technical assistance. Suggested improvements to the User Interface were to use less technical language. All participants who evaluated the PTS in this experiment were permanent wheelchair users. They stated that the use of this system would help them be more independent and worry less about the consequences of experiencing AD when performing typical ADL independently.

## VI. Discussion

Recognition and prevention of symptoms are critical to avoid escalation of AD in clinical and non-clinical settings. We have developed a user-friendly and intuitive physiological telemetry system using a commercially available smartwatch for tetraplegics to detect the onset of AD symptoms with high accuracy and low error rate of missing AD symptoms. This work is early translational research and aims to develop a prototype AD detection system for continuous monitoring that is efficient, usable, and reliable for future clinical studies involving a larger population of SCI users. The PTS would serve as a complementary tool to assist with an improved education of AD in individuals with SCI. It could also be used to help with management of AD for people in community settings.

### A. Using a Multimodal Approach to Detect AD Symptoms

Ambulatory blood pressure systems currently available to clinically detect the onset of AD for extended periods of time can be obstructive or intrusive, often obstructing activities of daily life [23]. Regular compression from the arm-worn blood pressure cuffs can lead to disturbances in sleep and can cause physical distress. Through the use of a commercially available, multimodal sensor smartwatch, it was possible to collect reliable physiological data for long periods of time with minimal intrusiveness into activities of daily life of users in community settings.

In prior studies, it was identified that the MS Band was able to reliably detect changes in the relevant physiological parameters needed for the PTS - GSR, ST and HR [14]. The MS Band was also well tolerated by participants for up to eight hours of continuous wear and reliably collected data during this time period. Although the MS Band was an order of magnitude less accurate compared to a conventional GSR recording system, the data collected was precise making it a viable replacement for the conventional system which comprises of electrodes worn on the body [14].

The PTS machine learning model can also be retrained to classify AD using alternate smartwatch systems. If other sensors prove to be more accurate in the detection of the relevant physiological parameters [37], we believe the machine learning model would result in similar or more accurate detection of AD symptoms.

### B. Physiological Parameter Identification for Machine Learning Model

The most common symptoms of AD are sweating above the level of injury as well as cold, clammy skin. This intuitively suggests that there would be observed changes in the GSR which changes due to the conductivity of the skin, as well as skin temperature. This validates the combination of GSR and skin temperature as the primary physiological parameters leading to the development of the most accurate and error free machine learning model.

While bradycardia is observed in individuals during onset of AD, tachycardia can also commonly occur [38]–[40]. This difference in the presentation of the heart rate during onset of AD may contribute to the lower accuracies and higher false negatives seen when a model is developed with a combination of GSR and heart rate.

### C. Clinical Relevance of Machine Learning Model Performance Measures

The optimal machine learning model for the detection of AD was one that produced the highest accuracy, sensitivity, and specificity. We prioritized the development of a machine learning model that not only was the most accurate but also had a low false negative error rate. A high false negative rate due to the model’s inability to detect AD symptoms when they occurred could lead to serious medical consequences, particularly if the PTS was being used as a training tool for newly injured individuals to recognize symptoms of AD. In contrast, the incidence of false positive errors would be more of an inconvenience to users rather than not detecting episodes of AD at all. Sensitivity and specificity in the detection of AD need to be balanced in order to ensure low false negative rates, even if it meant a slight increase in false positive rates and decrease in accuracy [41]. The model’s high AUC-ROC (0.94) demonstrated the model’s ability to detect the onset of AD symptoms with high accuracy with an inclination towards low false negative rates.

The occurrence of false positives can be attributed to several factors including participants underreporting instances of AD when they occurred. Self-reporting of AD by users was used as the ground truth for reliability. Since all participants had been injured for over three years and had experienced AD several times prior to participating in this study, we believe they are reliable source of information regarding their experiences with AD symptoms. However, a participant could have forgotten to report AD or missed the notification or been busy managing their AD symptoms, which may have led to a false positive. Some participants also reported changes in their threshold for detecting AD since their injury wherein they no longer associated mild AD symptoms with dangerous AD events. Despite participants being asked to report minor instances of AD symptoms, we believe the occurrence of false positives determined by the PTS was likely influenced by acclimatization to mild episodes of AD, such as when exercising or other nonemergent activities. Additionally, some of the false positives may have also occurred due to the onset of asymptomatic AD, which may have led to changes in physiology which went unnoticed by the participants [42], [43].

We specifically chose to use the presence of individual-reported AD symptoms as our ground truth for determining the accurate AD instead of changes in blood pressure measurements or other physiological markers. Though an increase in blood pressure is the objective measure for affirming the diagnosis of AD in a clinical setting, our goal was to develop an automated AD detection system for real-world monitoring of AD symptoms that would be practically useful for persons living with SCI. Symptomatic AD detection plays a crucial role in reinforcing techniques for training newly injured individuals to recognize AD and is the gold standard for AD management taught to newly injured individuals Whether an increase in blood pressure of 20 mm Hg (the clinical gold standard for AD diagnosis) causes symptoms that an individual tetraplegic would identify as a significant AD episode is also highly subjective.

### D. Usability of the PTS

The participants’ assessment of the PTS was favorable with a score of 86.2. The SUS is scaled from 0–100, with reviews of all studies utilizing the SUS since the inception of the scale [33] propose that products which are considered more user friendly often score in the high 70s to upper 80s [29]. The convenience and user friendliness of the system justifies the use of the PTS for further work involving AD detection in community settings.

### E. Future Work

In future studies, inclusion of heart rate measurements combined with an activity tracker may allow reduction of the false positive rate. Identifying the effect of exercise or other cardiovascular activities on the physiological parameters would allow the model to account these variations to normal activities. This would also confirm if the false positives originated from misclassification by the PTS or the under-reporting of AD by users.

During further studies we will also incorporate the use of cuff-based ambulatory blood pressure systems in participants with SCI to detect the onset of clinical AD as well as self-reported AD symptoms. This would help with characterizing asymptomatic AD as well as further validate the use of the PTS. Due to the low sampling rate of ambulatory blood pressure systems, the PTS must immediately record at the onset of symptoms.

## VII. Conclusion

In this paper we present a highly accurate and sensitive, user-independent Physiological Telemetry System which can detect the onset of AD symptoms. The system uses multimodal sensing data, including heart rate, galvanic skin response and skin temperature data collected from individuals with SCI to train SVM models. Experimental results indicated the model using GSR and skin temperature data had the highest accuracy to detect the onset of AD. A real-world community-based validation of this model with naïve tetraplegics revealed the universality and usability of the developed system for long-term monitoring.

The PTS can be used to assist tetraplegics in preventing the occurrence of AD symptoms and encourage individuals to practice better healthcare management to prevent future occurrences. It can be used as a training tool for newly injured individuals to familiarize themselves with the onset of AD symptoms, thus reducing risk of mortality. Since the PTS incorporates a mobile device, it is possible to automatically and unobtrusively transmit alerts of AD events to caregivers or medical professionals. This would promote greater self-sufficiency and independence among individuals with SCIs.
